# *TERT* promoter mutations and polymorphisms as prognostic factors in primary glioblastoma

**DOI:** 10.18632/oncotarget.4389

**Published:** 2015-06-22

**Authors:** Mohamed Ali Mosrati, Annika Malmström, Malgorzata Lysiak, Adam Krysztofiak, Martin Hallbeck, Peter Milos, Anna-Lotta Hallbeck, Charlotte Bratthäll, Michael Strandéus, Marie Stenmark-Askmalm, Peter Söderkvist

**Affiliations:** ^1^ Department of Clinical and Experimental Medicine, Linköping University, Linköping, Sweden; ^2^ Department of Advanced Home Care and Department of Clinical and Experimental Medicine, Linköping University, Linköping, Sweden; ^3^ Department of Clinical Pathology and Department of Clinical and Experimental Medicine, Linköping University, Linköping, Sweden; ^4^ Department of Neurosurgery and Department of Clinical and Experimental Medicine, Linköping University, Linköping, Sweden; ^5^ Department of Oncology and Department of Clinical and Experimental Medicine, Linköping University, Linköping, Sweden; ^6^ Department of Oncology, District Hospital, Kalmar, Sweden; ^7^ Department of Oncology, Ryhov Hospital, Jönköping, Sweden; ^8^ Department of Clinical Pathology and Clinical Genetics and Department of Clinical and Experimental Medicine, Linköping University, Linköping, Sweden

**Keywords:** *TERT* polymorphism, *TERT* promoter mutations, *IDH1* mutation, glioblastoma, IL-6

## Abstract

Telomerase reverse transcriptase (TERT) activity is up-regulated in several types of tumors including glioblastoma (GBM). In the present study, 128 primary glioblastoma patients were examined for single nucleotide polymorphisms of *TERT* in blood and in 92 cases for *TERT* promoter mutations in tumors. *TERT* promoter mutations were observed in 86% of the tumors and of these, C228T (−124 bp upstream start codon) was detected in 75% and C250T (−146 bp) in 25% of cases. *TERT* promoter mutations were associated with shorter overall survival (11 vs. 20 months *p* = 0.002 and 12 vs. 20, *p* = 0.04 for C228T and C250T, respectively). The minor alleles of rs2736100 and rs10069690 SNP's, located in intron 2 and the promotor regions, respectively, were associated with an increased risk of developing GBM (*p* = 0.004 and 0.001). GBM patients having both *TERT* promoter mutations and being homozygous carriers of the rs2853669 C-allele displayed significantly shorter overall survival than those with the wild type allele. The rs2853669 SNP is located in a putative Ets2 binding site in the promoter (−246 bp upstream start codon) close to the C228T and C250T mutation hot spots. Interleukin-6 (IL-6) expression regulated by *TERT* promoter status and polymorphism, what leads us to think that *TERT* and *IL-6* plays a significant role in GBM, where specific SNPs increase the risk of developing GBM while the rs2853669 SNP and specific mutations in the *TERT* promoter of the tumor lead to shorter survival.

## INTRODUCTION

Gliomas are the most common malignant brain tumors, where in adults primary glioblastoma (GBM) is the most aggressive. It generally appears in the sixth through eighth decades of life [1] and the number of patients is expected to increase with an aging population. Several prognostic and/or predictive factors for overall survival (OS) have been reported such as patient age, functional status, grade of resection, type of oncological treatment, and methylation status of the O(6)-Methylguanine-DNA methyltransferase (*MGMT*) gene promoter [2–4]. Mutations in the isocitrate dehydrogenase 1 (*IDH1)* and 2 (*IDH2)* genes, being markers for progression from lower grade glioma, have recently become clinical markers of predictive and prognostic importance [5]. Increased telomerase activity is one of the hallmarks of human tumors and *TERT* is frequently up-regulated in a variety of tumors including gliomas, preventing telomere shortening and inhibiting apoptosis and senescence [6, 7]. Somatic mutations in the coding region of *TERT* are infrequent in human tumors, but germ line genetic variants and somatic mutations in the *TERT* promoter is frequently found in human melanomas and also other human cancers and cell lines [8, 9]. Regulation of telomerase reactivation in tumor cells is complex and multifactorial and involves direct transcriptional activation as well as epigenetic regulation of *TERT* mRNA expression. Two somatic mutations, C228T and C250T located at −124 and −146 pb upstream from the ATG start site confer enhanced *TERT* promoter activity, by putatively generating consensus binding sites (GGAA) for ETS transcription factors within the *TERT* promoter region [8, 9]. These mutational hot spots are most frequently found in primary glioblastomas (82%) and oligodendrogliomas (78%), but are less common in secondary glioblastomas (35%) [10, 11]. In addition to somatic *TERT* promotor mutations, germ line genetic variants have been shown to predispose for breast, ovarian and bladder cancer [12, 13]. One common polymorphism in the promoter, rs2853669, is located in an existing Ets2 binding sequence (−246 bp upstream start codon), where the variant T-allele displays reduced transcriptional activity in luciferase reporter assays [13]. *TERT* rs2853669 has been shown to modulate both *TERT* expression and impact on prognosis in bladder cancer and GBM [13, 14].

McKay et al. published the first study indicating that the *TERT* rs2736100 polymorphism may contribute to an increased risk of lung cancer [15]. Since then, several research groups have reported associations between this SNP and cancer risk [16]. Genome wide association studies (GWAS) of glioma have shown that *TERT* is one of the genes that is strongly associated with glioblastoma [17–19].

To maintain telomere integrity, the protection of telomeres 1 (*POT1*) gene and the POT protein is regulating the substrate access for TERT, and thus responsible for limiting telomere elongation [18]. *POT1* has been identified as a susceptibility locus for GBM and mutations in the *POT* gene have also been reported [21]. Alternative lengthening of telomeres (ALT), involving both genetic and epigenetic mechanisms, has also been suggested as an alternate way to activate telomerase [22]. In hepatocellular carcinoma (HCC) the *POT1* rs7784168 SNP is significantly associated with a decreased risk of major vessel invasion and postoperative recurrence. Moreover, the *POT1* rs7784168 TC/CC genotype showed a significant effect with improved OS, and this suggests that the C allele of *POT1* rs7784168 may be associated with favorable prognosis for patients with HCC [23].

In addition to maintaining telomere integrity, the TERT protein presents additional functions and has been shown to interact with NFκB and co-activate the expression of several genes, including cytokines, such as IL-6 and TNFα, which are critical for inflammation and cancer progression [24–26].

In this study, we analyzed SNV's of the *TERT* and *POT1* genes in blood and the relation of *TERT* promoter mutations in the tumors to mRNA expression of TERT and pro-inflammatory cytokines and to overall survival.

## RESULTS

### Mutation analysis

**Figure 1:**
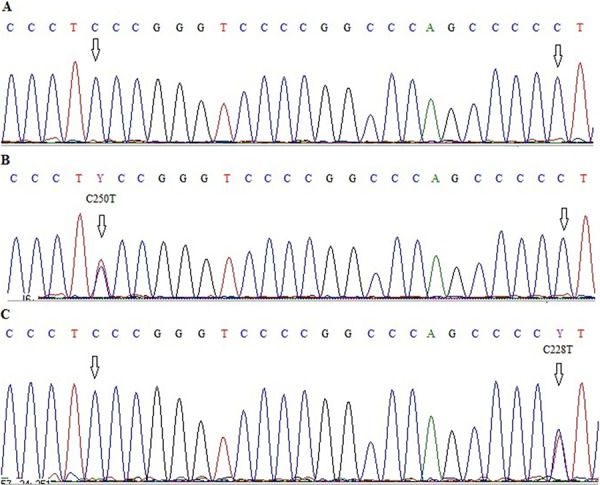
Electropherograms showing sequence of *TERT* promoter region with two hot-spot mutations C228T and C250T **A.** wild type C250 and C228, **B.** wild type C228 and C250T mutation, **C.** C228T mutation and wild type C250.

Somatic *TERT* promoter mutations were observed in 79/92 primary GBMs (85.9%). C228T was detected in 59 of these tumors (74.7%) and C250T in 20 (25.3%), the mutations being mutually exclusive. All mutations are heterozygous apart from one C228T mutation being homozygous, indicating a function as an oncogene (Figure 1).

**Figure 2:**
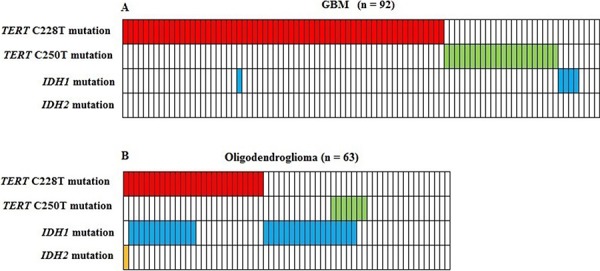
Correlation between *TERT* promoter mutations and IDH1/IDH2 mutations in 92 glioblastoma **A.** and 63 oligodendroglioma **B.** Five GBM patients had IDH1 mutations, but no mutations in IDH2 were observed. Only one GBM patient had both a C228T TERT promoter and an IDH1 mutation, revealing a significant inverse correlation (*p* < 0.001).

The frequency of *TERT* promoter mutations in an additional cohort of 63 oligodendroglioma was 54% (34/63), confirming earlier observations of lower frequency in a less malignant brain tumor subtype [10]. Mutation analysis of *POT1* as a potential alternative mechanism to activate telomerase, disclosed no mutations in our 92 GBM patient cohort. Five GBM patients (5.4%) had *IDH1* R132H mutations, but no mutations in *IDH2* (R172K) were observed. Only one GBM patient had both a C228T *TERT* promoter and an *IDH1* mutation, revealing a significant inverse correlation (*p* < 0.001, Figure 2).

The mean age at GBM diagnosis was significantly higher in patients with a *TERT* promoter mutation than without, 63.2 ± 8.64 versus 53.15 ± 16.4 (*p* = 0.001) and no difference was observed for the two different mutations.

### *TERT* polymorphisms genotyping

Blood DNA samples were available for 128 GBM patients that were genotyped for four *TERT* SNPs; rs2853669 (T > C) located in the promoter region, rs2736100 (A > C), rs10069690 (C > T) and rs4246742 (T > A) located in introns 2, 4 and 8 respectively. Two of these SNPs, rs2736100 and rs10069690 are in LD *(D*´ = 0.83). Multivariate logistic regression analysis showed that homozygosity for the C variant of rs2736100 and T for rs10069690, were associated with a strong significantly increased risk of developing primary glioblastoma compared to the corresponding wild type AA and CC genotypes (OR = 2.16, 95% CI 1.23 – 3.8, *p* = 0.004 and OR = 3.12, 95% CI 1.58 – 6.17, *p* = 0.001, respectively (Table 1). The AC and CT genotypes displayed an intermediate but still significantly increased risk (OR = 1.55, 95% CI 0.92 – 2.16, *p* = 0.05 and OR = 1.82, 95% CI 1.22 - 2.72, *p* = 0.002). Multivariate logistic regression analysis in the set of the 63 oligodendroglioma did not reveal any association with rs2736100 or rs10069690 SNPs (OR = 1.37, 95% CI 0.64 – 2.86, *p* = 0.27 and OR = 1.87, 95% CI 0.68 – 5.12, *p* = 0.17, respectively).

**Table 1:** Genotype distribution of the different polymorphisms in GBM patients and normal control population and their association with GBM susceptibility

Gene	Polymorphisms	GBM *n* (%)	Controls *n* (%)	OR (95 % CI)	*p*-Value
*TERT*	rs2853669				
	TT	69 (53.90)	373 (47.88)		
	TC	48 (37.5)	341 (43.77)	0.76 (0.51 – 1.13)	0.10
	CC	11 (8.59)	65(8.34)	0.91 (0.45 – 1.82)	0.47
	TC + CC	59	406	0.78 (0.54 – 1.14)	0.12
	**Total**	**128**	**779**		
	**rs2736100**				
	AA	21 (16.40)	201 (25.5)		
	AC	66 (51.56)	406 (51.53)	1.55 (0.92 – 2.16)	**0.05**
	CC	41 (32.03)	181 (22.97)	2.16 (1.23 – 3.8)	**0.004**
	AC + CC	107	587	1.74 (1.06 – 2.86)	**0.01**
	**Total**	**128**	**788**		
	**rs10069690**				
	CC	47 (36.72)	409 (53.32)		
	CT	67 (52.34)	319 (41.60)	1.82 (1.22 – 2.72)	**0.002**
	TT	14 (10.93)	39 (4.68)	3.12 (1.58 – 6.17)	**0.001**
	CT + TT	81	358	1.96 (1.33 – 2.89)	**0.0003**
	**Total**	**128**	**767**		
	**rs4246742**				
	TT	82 (64.06)	520 (66.41)		
	TA	43 (33.59)	240 (30.66)	1.13 (0.76 – 1.69)	0.29
	AA	03 (2.34)	23 (2.93)	0.82 (0.24 – 2.80)	0.52
	TA + AA	46	263	1.10 (0.75 – 1.63)	0.33
	**Total**	**128**	**783**		
*POT1*	**rs7784168**				
	TT	61 (47.65)	318 (42.74)		
	TC	54 (42.18)	349 (46.9)	0.80 (0.54 – 1.19)	0.16
	CC	13 (10.15)	77 (10.34)	0.88 (0.46 – 1.68)	0.41
	TC + CC	67	426	0.81 (0.56 – 1.19)	0.17
	Total	**128**	**744**		

*TERT* rs2853669 (T > C) is located at −246 upstream the ATG start codon, in close proximity to the two somatic hot spot mutations C228T and C250T. The variant allele did not show any association with susceptibility for GBM (OR = 0.91, 95% CI = 0.45 – 1.82, *p* = 0.47), which is in accordance with a suppressed transcriptional activity reported for this variant (CC) compared to the wild type variant (TT). Nevertheless rs4246742 showed no association with an increased risk for GBM or effect on survival.

### Overall survival of GBM patients

**Figure 3:**
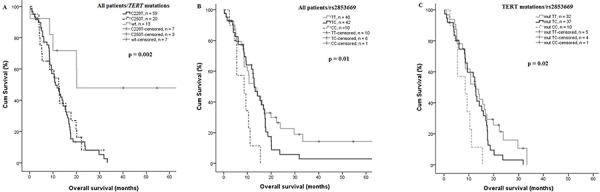
Effect of *TERT* promoter mutations on OS **A.** *TERT* promoter mutations (C228T and C250T) was significantly associated with a shorter OS, the mean OS was 11 vs. 20 months, *p* = 0.002, and 12 vs. 20 months *p* = 0.04, for C228T and C250T, respectively (*n* = 92). **B.** GBM patients homozygous (CC) rs2853669 showed a significantly shorter OS compared with heterozygous (TC) and (TT) 8.2 months vs 15.7 and 24.2 months, *p* = 0.007 and 0.02 respectively (*n* = 92). **C.** *TERT* mutant patient homozygous (CC) showed a shorter OS compared to *TERT* mutant heterozygous and non-carriers of the C-allele, 8.2 months vs. 12.3 and 14.8, *p* = 0.02 and 0.023 respectively (*n* = 79).

The *TERT* promoter mutations C228T and C250T were significantly associated with shorter survival in univariate analysis (median 11 vs. 20 months *p* = 0.002 and 12 vs. 20 months, *p* = 0.04 for C228T and C250T, respectively) compared to wild type tumors (Figure 3A). Interestingly, the impact of the promoter mutations on OS was influenced also by the rs2853669 genotype, where the variant allele destroys an Ets2 binding site. Patients being homozygous carriers of the C variant of rs2853669 SNP showed a significantly reduced OS, 8.2 months compared to TC or TT, 15.7 and 24.2 months, respectively (*p* = 0.007 and 0.02, Figure 3B). Stratification of *TERT* promoter mutant patients according to presence of the polymorphic rs2853669 SNP showed a significantly shorter OS in patients harboring *TERT* mutations and being CC carriers, 8.2 months compared to heterozygous TC carriers, 12.3 months (*p* = 0.023) and wild type TT, 14.8 months (*p* = 0.02 Figure 3C). The Prognostic impact of age at diagnosis was analyzed for the entire GBM patient cohort using 63 years as a cut-off, being the median age of the cohort. A significantly reduced OS was shown for older patients (>63 years) *p* = 0.05. The impact of the *TERT* promoter mutations status on patient survival was also analyzed in age stratified patient subgroups, and a significantly reduced OS was observed in the subgroup older than 63 years (*p* = 0.02, Supplementary Figure S1), although the patient groups are small (4 patients being wild type). In contrast, rs10069690 and rs2736100, that showed a risk for GBM (*p* = 0.004 and 0.001 respectively), had no effect on survival in our GBM cohort, the same as for rs4246742.

To evaluate whether *TERT* promoter mutations are independent prognostic markers compared to other risk factors for survival in the 92 GBM patients, multivariate Coxregression analysis was performed including the covariates age, gender, *TERT* promoter mutations, *IDH1* mutation and *TERT* promoter mutations combined with rs2853669. The Coxregression analysis showed that *TERT* promoter mutations are independent predictors for decreased survival (*p* = 0.004 and *p* = 0.04 for C228T and C250T, respectively), in addition to higher age (*p* = 0.01). The rs2853669 CC genotype was also identified as an independent predictor of short patient survival in *TERT* promoter mutated patients (*p* = 0.002, Table 2).

**Table 2:** Cox regression of overall survival

*Covariates*	HR	95 % CI	*p*-Value
Age	1.03	1 – 1,06	**0.01**
Gender^a^	0.94	0.61 – 1,58	0.98
C228T mutation^b^	4.04	1.55 – 10,51	**0.004**
C250T mutation^b^	3.7	1.3 – 10,51	**0.04**
TERT mut+rs2853669 TT			
TERT mut+rs2853669 TC^c^	4.7	1.92 – 17.16	**0.01**
TERT mut+rs2853669 CC^d^	10.72	2.47 – 46.50	**0.002**
IDH1 mut^e^	1	0.26 – 3.83	0.99

a Female compared to male gender,

b Mutated compared to non-mutated,

c Mutated+ rs2853669 TC compared to Mutated rs2853669 TT,

d Mutated+ rs2853669 CC compared to Mutated+ rs2853669 TT,

e IDH1 mutated compared to non mutated.

### Gene expression

**Figure 4:**
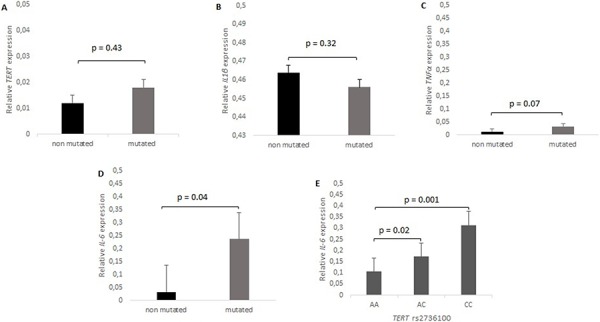
mRNA expression levels of TERT, IL-6, TNFα and IL-1β, in relation to TERT promoter mutation status **A.** TERT is 1.68 (*p* = 0.43) fold higher in GBM *TERT* mutants compared to wild type. **B.** No difference of IL-1β mRNA expression between mutated and wild type (*p* = 0.32). **C.** TNFα expression is 2.61 fold higher in TERT mutated tumors (*p* = 0.07). **D.** IL-6 is enhanced 7.39 fold in TERT mutated compared to wild type GBM (*p* = 0.04), and **E.** IL-6 was 2.95 and 1.63 fold higher in patient with CC and CA respectively compared to patient with AA rs2736100 genotype, *p* = 0.001 and 0.02 respectively.

Besides telomere elongation, telomerase harbor other functions that may contribute to carcinogenesis. The TERT protein has been suggested to co-activate NFκB to stimulate gene transcription and enhance expression of *e.g* IL-6, TNFα and other pro-inflammatory cytokines. Relative mRNA expression analysis was performed on available tumor tissue from 34 patients, four being wild type and thirty four mutated in the *TERT* promoter. The relative mRNA expression for IL-6 was significantly increased (*p* = 0.04, Figure 4C), but no significant differences in mRNA expression were apparent for TNFα, IL-1β or *TERT* (Figure 4A, 4D, 4F). No significant differences in mRNA expression was identified in the *TERT* promoter mutated group compared with the wild type group (*p* = 0.43, Figure 4A), whereas *TERT* was significantly increased in tumors harboring C228T (4 patients) compared to C250T mutations (28 patients) (*p* = 0.0003). We have shown that only *TERT* rs2736100 modify NFκB mediated expression of IL-6 in GBM, to be significantly increased in patients with CC or AC genotypes compared to those with AA genotype (*p* = 0.001 and 0.02, respectively). Figure 4E. Dividing the patients according to the IL-6 median mRNA expression level (0.081) there was no correlation between IL-6 mRNA expression levels and survival (*p* = 0.97), 17 months for patients below the IL-6 median and 16 months for those above.

## DISCUSSION

Telomerase is involved in many fundamental cellular processes, such as cellular senescence and immortalization, epigenetic programming and metabolism [27]. In this work we screened a set of 128 GBM patients regarding *TERT* SNVs as risk factors for developing GBM. We report a significant association of certain *TERT* SNPs with risk of developing glioblastoma. Four *TERT* SNPs were examined in white blood cells DNA. We found that *TERT* SNPs rs2736100 and rs10069690 located in intron 2 and 4 respectively were associated with increased risk of glioblastoma, but not for oligodendroglioma. *TERT* rs2736100 has previously been described in several GWAS to be associated to increased risk for glioma, [16, 28–29]. Our results corroborate with the findings by Simon M et al. (2010) that the frequency of rs2736100 risk genotype (CC) correlates to high-grade disease of glioma (GBM) as compared to lower grades *e.g* oligodendroglioma [30]. A meta-analysis including 9411 cases and 13708 controls showed that *TERT* rs2736100 was significantly associated to GBM also to astrocytoma and oligodendroglioma [29].

Despite their association with increased risk of GBM, Kaplan-Meier estimates showed no evidence for an independent relationship between genotype of rs2736100 or rs10069690 and OS. On the other hand, rs2853669 that is not related to a risk for developing GBM, does significantly influence OS in *TERT* promoter mutated GBM patients. This SNP has been shown to interfere with an Ets2 binding site in the *TERT* promoter, and Xu and collaborators (2008) have shown that the minor genetic variant (the C-allele) inhibits c-Myc binding to the E-box consensus site that is present with the major T-variant allele in breast cancer cells [31]. In addition, the rs2853669 CC genotype has been shown to influence telomerase activity (TA) and telomere length maintenance in non-small cell lung cancer [32] and bladder cancer [13]. Several studies showed that the telomerase activity and *TERT* mRNA expression levels were significantly higher in mutated GBM as compared to those with wild-type *TERT* promoter [11, 14, 36]. Further, Labussière et al. (2014) have shown that GBM patients harboring the variant allele (CC + TC) rs2853669 showed a two-fold reduction in *TERT* expression as compared with TT homozygote [33].

Survival analysis revealed that GBM patients with *TERT* mutations have significantly shorter OS than patients without mutations (*p* = 0.002) in accordance to other reports [10, 14, 34]. This is in contrast to Naosuke et al., who found a significant effect in primary and secondary GBMs combined (*p* = 0.015) but not in 167 primary GBMs alone (*p* = 0.22) [35]. Our results confirm the findings recently reported concerning the effect of *TERT* mutation and the rs2853669 C-variant on overall survival, where patients carrying both an activating *TERT* promoter mutation and being homozygous for the rs2853669 C variant have a reduced mean OS [14]. In contrast, Simon et al. found that poor survival was restricted to patients without the variant rs2853669 C-allele in GBM, [34] while in bladder cancer Rachakonda et al. (2013) have shown that the presence of the variant rs2853669 C-allele was beneficial in mutant and detrimental in a wild type *TERT* promoter background [13].

*IDH1/IDH2* mutations are known to be diagnostic molecular markers of secondary glioblastoma [37]. Similar to other studies, GBM patients with *IDH1* mutations were on average 15 years younger than those without *IDH1* mutations 47 ± 7.2 versus 62.6 ± 1.0 years. Only one *IDH1* mutant patient carried a *TERT* mutation. Nobusawa et al. (2009) identified 14 of 407 primary GBM (3.4%) with *IDH1* mutations and these patients were on average 10 years younger. They show that GBM with *IDH1* mutation diagnosed as primary had clinical and genetic profiles similar to those of secondary glioblastomas, suggesting that they may have rapidly progressed from a less malignant precursor lesion that escaped clinical diagnosis and were thus misclassified as primary [37].

In addition to *TERT*, *POT1* is known to negatively regulate telomere length by directly inhibiting telomerase activity, or by controlling telomeric DNA substrate access to telomerase in human cells [20]. Little is known about the impact of *POT1* genetic variation on telomere function and cancer risk. Ramasay and collaborators found twelve somatic mutations (5%) in *POT1* of chronic lymphoid leukemia (CLL) cases, nine detected in the N-terminal OB domain for *POT1* and three mutations leading to a truncated protein. The *POT1* mutations are hypothesized to favor the acquisition of malignant features in CLL [38] and may represent a possible novel approach for the clinical management of this disease. Two previous studies in breast and lung cancer in Polish and Chinese populations did not observe any association between *POT1* polymorphism and disease risk [39, 40]. In our study in GBM we found no association between *POT1* polymorphism and OS or risk of tumor development and no *POT1* mutations were detected in our cohort.

Previous studies have shown that *TERT* promoter mutations result in a significant increase in *TERT* mRNA expression and telomerase activity in tumors compared to wild type. Nevertheless, in the tumors where RNA was available, we were unable to demonstrate a difference in our study, possibly due to a low number of wild type samples (*n* = 4) compared to 34 mutated samples. The influence of an inflammatory microenvironment has long been considered important in the initiation and progression of GBM [26]. However, the success of developing therapeutic approaches to target inflammation for GBM therapy has yet been limited [26]. Ghosh et al. (2012) have shown in gene expression analysis that leukemic cells from AML, ALL or CML patients display reduced levels of IL-6, a target gene of the transcription factor NFkB, when treated with the telomerase inhibitor MST-312. These results reiterate that inhibiting telomerase activity in cancers could be an effective means of blocking NFkB target genes that promote inflammation and malignant transformation [24]. Thus, this suggests that the *TERT* mutations and increased telomerase activity may maintain a tumor phenotype by stimulating an inflammatory response and angiogenesis as suggested by the increased IL-6 levels [24]. In our GBM cohort a significantly increased expression of IL-6 but not of TNFα or IL-1β was detected according to *TERT* promoter mutation status. Further, IL-6 plays critical roles in the progression of non-small cell lung cancer (NSCLC) [25], and the increased serum concentrations in patients are associated with advanced tumor stages of several tumors (e.g., multiple myeloma, renal cell carcinoma, prostate cancer, breast and ovarian cancer) [41, 42]. As *TERT* has been shown to modify the expression of numerous genes including IL-6 by co-activating NFκB transcription [41, 42]. Wang shows that *TERT* rs2736100 A/C polymorphism affect the expression of IL-6 and IL-6 levels was significantly elevated in NSLC subjects with the CC genotype, compared to those with AA (*p* < 0.01) [25].

Our study shows similar findings, IL-6 mRNA levels were significantly increased in patients with CC or AC genotypes compared to those with the AA genotype (*p* = 0.001 and 0.02, respectively. In the same context, Rolhion and collaborators found a significantly increased expression of IL-6 among 43 GBM compared to 16 non GBM brain tumors (astrocytomas, pilocytic astrocytomas, oligodendrogliomas and oligoastrocytomas) (*p* < 0.001), and suggest that IL-6 may play a central role in GBM behavior, which may serve as a suitable new potential target in the treatment of glioblastomas as well as a marker in glioma classification [43]. In our study the increased IL-6 levels are related to the *TERT* promoter mutation status and polymorphism. Cheng-Yi et al. demonstrated a trend to a difference on OS between patients with IL-6 positive immunohistochemistry (5/11) in GBM patients, 7 months median survival versus 16 months, *p* = 0.075 [44], whereas in our cohort the median OS for patients with low IL-6 mRNA expression (< median 0.081) was 17 months in relation to those with high IL-6 expression levels (>0.081), that had an median OS of 16 months (*p* = 0.97).

In conclusion, our results shows that certain *TERT* SNPs are associated with an increased risk of developing GBM, and an association between somatic *TERT* promoter mutations also results in a reduced OS. An additional negative effect is observed for simultaneous presence of *TERT* promoter mutations and the presence of the variant C-allele of rs2853669 SNP. Our results also indicate that the *TERT* rs2853669 polymorphism, *TERT* promoter mutations and IL-6 expression have potential to become prognostic markers of survival in GBM, which may further aid clinicians in treatment decisions.

## MATERIALS AND METHODS

### Study subjects

This study was approved by the Regional Ethics Committee at Linköping University, Linköping, Sweden. One hundred twenty eight patients diagnosed with primary glioblastoma were included in this study blood DNA were used for genotyping. In 92 cases, tumor tissue was available for mutation screening and in 52 of these cases both blood and tumor tissue was available. The clinical information, including age, gender and survival data, was obtained from the Swedish Cancer registry. The mean age of patients at diagnosis was 62.5 ± 11.0 years. The male to female ratio was 1.3:1. A control population for analyzing the role of SNPs for primary glioblastoma comprised 788 healthy individuals (50% women, 50% men) with mean age of 54 ± 17 years, randomly collected from the population register from the same geographic region as the patients during the years 1998 to 2000. For further comparison we also analyzed 63 oligodendrogliomas.

### DNA extraction

Genomic DNA was extracted from blood samples and frozen tumors tissues. From blood samples DNA was extracted using Maxwell^®^ 16 Blood DNA Purification Kit according to the supplier's recommendations (Promega, Madison, WI, USA). Extraction of DNA from frozen tissue, was performed with, AllPrep DNA/RNA Mini Kit (Qiagen, USA).

### RNA extraction and real-time quantitative PCR

RNA was available for sixty two GBM samples, 4 wild type and 34 containing somatic *TERT* promoter mutations, and was investigated for *TERT* mRNA expression. Total RNA from each sample was reversely transcribed into cDNA with MaximaR First Strand cDNA synthesis Kit for RT-qPCR (Fermentas, St Leon-Rot, Germany) according to the supplier´s instructions. The relative mRNA expression of *TERT*, *IL-6, IL-1β* and *TNFα* was determined by real-time PCR [7900HT Fast Real-Time PCR System (Applied Biosystems)], which was performed with the TaqMan^®^ Fast Universal PCR Master Mix (Applied Biosystems) and cDNA-specific primer/probe mixes for *TERT*, *IL-6, IL-1β* and *TNFα* (Hs00972656_m1, amplicon length 79 bp, Hs00985639_m1, amplicon length 66 bp, Hs01555410_m1, amplicon length 91 bp and Hs01113624_g1, amplicon length 143 bp, respectively) (Applied Biosystems). The relative expressions of the genes were normalized to the expression of two control genes *β-glucuronidase* (*GUSB*) (4333767F, amplicon length 81 bp) and *Hypoxanthine-guanine phosphoribosyltransferase 1* (*HPRT1*) (4333768F, amplicon length 100 bp) (Applied Biosystems) and the GBM wild type group was considered as calibrator. The PCR reactions consisted of 20 ng cDNA, 5 μl of TaqMan Fast Universal Master mix (2X), 0.5 μl primer-probe assay mix in a final volume of 10 μl. The cycling conditions were as follows: 20 s in 95°C for denaturation and then 40 cycles consisting of 3 s at 95°C, 30 s at 60°C. All samples were run as duplicates. Calculation of normalized gene expression was based on established methods the so-called ΔΔC_T_ method. ΔC_T_ was calculated from subtracting mean C_T_ value of *HPRT1* and *GUSB* from mean C_T_ value of *TERT*. The 2^−ΔCT^ formula was used to calculate final relative expression of TERT, IL-6, IL-1β and TNFα.

### *TERT* SNP genotyping

The SNPs were chosen on the basis of previous reports of their association with the risk for cancer and a comprehensive tag SNP approach. The rs2853669, rs2736100 and rs4246742 in *TERT*, and rs7784168 in *POT1* were genotyped using TaqMan^®^ SNP Genotyping assays (C_8773290_10, C_1844009_10, C_11772271_20, C_103023_10 respectively). Total volume per well was 10 μl composed of 5 μl TaqMan^®^ Universal PCR Master Mix (2X), 0.25 μl assay mix (40X), including the two allele-specific TaqMan^®^ MGB probes, 3.75 -μl H_2_O and 20 ng of DNA. All analyses were performed in ABI Prism 7500 or 7900 Sequence Detection System, using the SDS 1.3 and 2.4 software for allelic discrimination (Applied Biosystems) respectively.

The rs10069690 was genotyped in glioblastoma patients and healthy controls by pyrosequencing. A DNA fragment was amplified by PCR. Each PCR reaction consisted of 5 mM My Taq Buffer (Bioline, UK), 1 U My Taq DNA polymerase, 1 μM of each primer (Supplementary Table S1) and 20 ng of template DNA in a final volume of 15 μl. The cycling conditions were 95°C for 2.5 min, 35 cycles of 95°C denaturation for 30 s, 66.1°C annealing at 30 s and extension at 72°C for 30 s. Successful PCR was determined by 1.5% agarose gel electrophoresis. The genotypes were assessed on a PSQ96MA instrument (Qiagen, Sweden) as previously described [45]. In brief, the amplified and biotinylated PCR product was isolated with a Vacuum Prep Workstation (Qiagen, Sweden). The sequencing primer 5′-gggtgaggtggacaga-3′ was annealed to the single-stranded DNA template for 2 min at 80°C. The plate was then transferred to the PSQ96MA instrument and sequencing was performed with the following dNTP dispensing order: cgt agc tgc.

### Mutation analysis

The *TERT* core promoter was amplified using MyTaq™ DNA polymerase (Bioline, USA) and primers found in literature [10, 46]. PCR was performed in a total volume of 15 μl, containing 5 mM My Taq Buffer (comprise 5 mM dNTP and 15 mM MgCl_2_), 1 μM of each primer (Supplementary Table S1), 1 U MyTaq™ DNA polymerase and 20 ng of template DNA. PCR products were purified with ExoSap-IT (GE Healthcare, USA), and standard Sanger sequencing was performed according to BigDye 3.1 protocol (Applied Biosystems, USA) and capillary electrophoresis on ABI 3500 Genetic Analyzer (Applied Biosystems, USA). PCR and mutation analysis of *POT1* were performed for the exons previously shown to harbor mutations (exons 5–10, 18) according to the same protocol as for *TERT.* Primers are listed in Supplementary Table S1.

### *IDH1* and *IDH2* exon 4 mutations

DNA fragments spanning exons 4 of *IDH1* and *IDH2*, previously identified as hot spots for mutations in these genes, [37] were amplified by PCR. Primer sequences for PCR are listed in Supplementary Table S1. Both PCR reactions were performed according to the same protocol as for *TERT* promoter amplification except the annealing temperature was 55°C.

### Statistical analysis

The genotype distributions in controls was tested and found to be in Hardy-Weinberg equilibrium. The association between each SNP and risk of glioblastoma was assessed by Odds ratios (OR) with 95% confidence intervals (CI). Student's *t*-test was performed to compare mean age of the patients with and without *TERT* promoter mutations. Bonferroni-Holm correction was used in our statistical analysis. For survival analysis Kaplan-Meier curves were generated and tested for significance by the log-rank test. OS was the time elapsed from diagnosis of primary glioblastoma to the date of death. Multivariate Cox regression models were applied to assess the impact of *TERT* promoter mutations on glioblastoma patient's survival. Statistical analysis was performed with SPSS Statistics 21 (IBM Corporation, Somers, NY) and Epi info7. *P* < 0.05 was considered as statistically significant. Linkage Disequilibrium (LD) between polymorphisms of the TERT gene were examined by pair-wise comparisons of D'-values using HaploView software version 4.2 (Broad institute, Boston, MA).

## SUPPLEMENTARY FIGURE AND TABLE


