# Supplementary material for: TERT promoter mutations and polymorphisms as prognostic factors in primary glioblastoma
Source: Oncotarget. 2015 Jun 22;6(18):16663–73. doi: 10.18632/oncotarget.4389 (PMC4599297; doi:10.18632/oncotarget.4389)
Supplement: Supplementary file 1 [file oncotarget-06-16663-s001.pdf]

SUPPLEMENTARY FIGURE AND TABLE

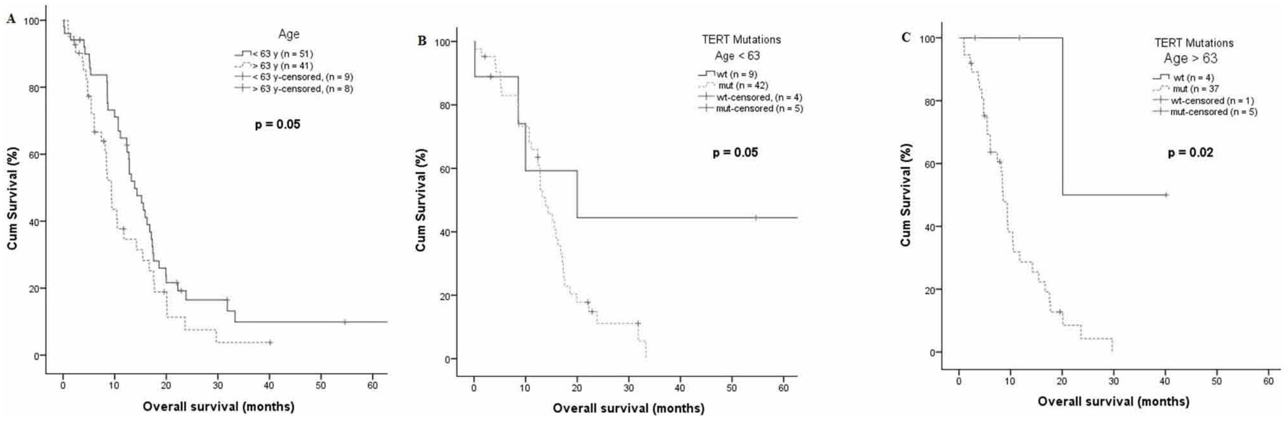

Supplementary Figure S1: The prognostic impact of median age at diagnosis (<63y/ ≥63y) was analyzed for the entire GBM patient cohort A. A significantly reduced OS was shown for older patients (>63 years) $p = 0.05$. The impact of the *TERT* promoter mutation status on patient OS was analyzed in age stratified patient subgroup **B. & C.** A significantly reduced OS was observed in the subgroup older than 63 years ($p = 0.02$).

Supplementary Table S1: *TERT, POT1* and IDH1/2 primers

	Forward primer	Reverse primer
TRET promoter	GGCCGATTCGACCTCTCT	AGCACCTCGCGGTAGTGG
TERT rs10069690	biotin-TCCTGGCCGCATGTGTGT	AGAGTGTGGGGTGAGGTGGA
POT1-EX5	AGCATGTAATCACATTGGAGGT	TCTATGTGTGTGGCATATACAGG
POT1-EX6	CATGGATTTGCTGCTAATATGAT	TGGAACCGTGTTCCTAAATC
POT1-EX7	TGAAAGCCAAGAAAATGTCTC	TGAAAAGCTTGCTGTCATGT
POT1-EX8	TTCCAGTTTCTTTGGTTCGT	CACAGCATGCTTTATCTCATCA
POT1-EX9	TGCCAATATTCAGAGGCATAAG	AAAAATTTACATGAGCAAAAATCAC
POT1-EX10	ATTGGCCCCATATTGGTTAT	GCACAAAAGGCTAGGGAACT
POT1-EX18	TCTAGATTATCTTTTAATTGGACA	ACTTCTAAAAGTCCACAGAGTACA
IDH1	CTCAGAGCCTTCGCTTTCTG	CACATACAAGTTGGAAATTTCTGG
IDH2	GGGGTTCAAATTCTGGTTGA	CTAGGCGAGGAGCTCCAGT
